# Supplementary material for: Pregnancy outcomes in women with vitiligo: A Taiwanese nationwide cohort study
Source: PLoS One. 2021 Mar 22;16(3):e0248651. doi: 10.1371/journal.pone.0248651 (PMC7984608; doi:10.1371/journal.pone.0248651)
Supplement: Attachment — Submitted filename: Reviewer opinion and response.doc [file pone.0248651.s001.doc]

**Editor(s)' and Reviewer(s)' Comments to Author:**

**Editor**

Comments to the Author

1. Please ensure that your manuscript meets PLOS ONE's style requirements, including those for file naming.

**Response**:

Thanks for your kind comment and reminder. The article has been sent for professional English editing and the certificate has been provided along with this revised manuscript.

2. In the ethics statement in the manuscript and in the online submission form, please provide additional information about the patient records used in your retrospective study, including: a) whether all data were fully anonymized before you accessed them; b) the date range (month and year) during which patients' medical records were accessed. If patients provided informed written consent to have data from their medical records used in research, please include this information.

**Response**:

Thanks for your kind comment and reminder. All data is governed by the Taiwan National Health Insurance Research Database, and it was fully anonymized information, which does not affect the welfare and right of the subjects. Thus, the Institutional Review Board of Tri-Service General Hospital approved our study, and the informed consent from our participants was waived.

3. Your ethics statement should only appear in the Methods section of your manuscript. If your ethics statement is written in any section besides the Methods, please delete it from any other section.

**Response**:

Thanks for your kind comment and reminder. We revised our article accordingly.

4. We suggest you thoroughly copyedit your manuscript for language usage, spelling, and grammar. If you do not know anyone who can help you do this, you may wish to consider employing a professional scientific editing service.

Upon resubmission, please provide the following:

1. The name of the colleague or the details of the professional service that edited your manuscript
2. A copy of your manuscript showing your changes by either highlighting them or using track changes (uploaded as a *supporting information* file)
3. A clean copy of the edited manuscript (uploaded as the new *manuscript* file)

**Response**:

Thanks for your kind comment and reminder. The article has been sent for professional English editing and the certificate has been provided along with this revised manuscript.

5. Thank you for stating the following in the Acknowledgments/Funding sources Section of your manuscript:

[This study was supported by grants from Tri-Service Hospital Research

Foundation (TSGH-B-109-010, TSGH-D-109-050, and 801GB110220).]

We note that you have provided funding information that is not currently declared in your Funding Statement. However, funding information should not appear in the Acknowledgments section or other areas of your manuscript. We will only publish funding information present in the Funding Statement section of the online submission form.

Please remove any funding-related text from the manuscript and let us know how you would like to update your Funding Statement. Currently, your Funding Statement reads as follows:

[The funders had no role in study design, data collection and analysis, decision to

publish, or preparation of the manuscript.]

Please include your amended statements within your cover letter; we will change the online submission form on your behalf.

**Response**:

Thanks for your kind comment and reminder. We revised our article accordingly.

**Reviewer**

Comments to the Author

1. The study of the relations between pregnant outcomes and vitiligo is quite rare, this research points out some unknown features by applying large scale research database.

**Response**:

Thanks for your kind comment. The purpose of our study is to emphasize the importance of vitiligo in pregnant women. Out of personal discussion, most obstetricians would check whether pregnant patients were previously diagnosed as SLE or Sjögren’s syndrome, but vitiligo which is also an autoimmune disease seldom drew their attention. Besides, our study also revealed the importance of disease control with systemic treatment to reduce the risk of miscarriage.

2. The Taiwan National Health Insurance (NHI) and the National Health Insurance Research Database (NHIRD) should be different. Are there any references indicating the coverage rate (>99% in the text) and the reimbursement system of NHI? Further, how it works between the NHI and NHIRD? Third, the abbreviation of NHIRD should be used when it first appeared in the manuscript.

**Response**:

Thanks for your kind comment and reminder. Taiwan National Health Insurance (NHI) is a single-payer compulsory social insurance. This system promises equal access to healthcare for all citizens, and the coverage had reached 99% [1]. NHIRD, the large computerized databases, derived from the National Health Insurance Administration (the former Bureau of National Health Insurance, BNHI), Ministry of Health and Welfare (the former Department of Health, DOH), Taiwan and maintained by the National Health Research Institutes, Taiwan, are provided to scientists in Taiwan for research purposes. The data in NHIRD is fully anonymized linked information, including outpatient care, inpatient care, emergency care, treatment procedures, and prescription drugs.

3. As mentioned in the study limitation section, the extend and severity of the vitiligo is difficult to be defined, therefore, the treatment of choice remained controversy. Topical agents have longstanding been used as the first line therapy, why only systemic treatments were regarded as preconception treatment? Consequently, should the 800 cases who were assigned as “without treatment” be “real non treatment” or “topical treatment”? Since those cases without treatment also remained controversy, the term used in the table 3 should be revised, and the conclusion also should be reconsidered accordingly.

**Response**:

Thanks for your kind reminder. As your comment, topical agents have longstanding been used as the first line therapy. In this study, we want to know if the systemic treatment before pregnancy decreases the risk of abortion or not, and we revised our article accordingly, including manuscript and tables.

4. As for the comorbid diseases, the SLE/Sjogren’s syndrome/RA were excluded for the enrollment in the beginning, so how to explain why those diseases still existed in the abortion group of vitiligo patients? Furthermore, immunosuppressive agents such as methotrexate and azathioprine are not common as the systemic medications for vitiligo prescribed by dermatologists, is there any explanation?

**Response**:

Thanks for your kind comment. This is a retrospective cohort study ranging from January 1^st^, 2000 to December 31^st^, 2015. SLE/Sjogren’s syndrome/RA were excluded for the enrollment in the beginning, and we rechecked our enrolled data that no one was diagnosed of SLE/Sjogren’s syndrome/RA before their first pregnancy. We kept following the medical records after their first pregnancy and found the increased risk of SLE/Sjogren’s syndrome/RA in patients with vitiligo who experienced abortion. Besides, steroid phobia is a common problem in my country. Some patients rejected any treatment containing steroid, including topical and oral agents. At that time, we will arrange phototherapy and/or prescribe topical calcineurin inhibitors and/or oral immunosuppressive agents, such as methotrexate and azathioprine, to control their vitiligo disease activity.

**Reference**

1.	Wu T-Y, Majeed A, Kuo KN. An overview of the healthcare system in Taiwan. London journal of primary care. 2010;3(2):115-9.
