# Supplementary material for: Pregnancy outcomes in women with vitiligo: A Taiwanese nationwide cohort study
Source: PLoS One. 2021 Mar 22;16(3):e0248651. doi: 10.1371/journal.pone.0248651 (PMC7984608; doi:10.1371/journal.pone.0248651)
Supplement: Attachment — Submitted filename: Reviewer opinion and response.doc [file pone.0248651.s002.doc]

**Editor(s)' and Reviewer(s)' Comments to Author:**

**Editor**

Comments to the Author

1. Please remove results from Discussion.

**Response**:

Thanks for your kind comment. The results were removed from discussion in the revised article accordingly.

2. Odds ratios among different studies (or models) should never be compared in terms of magnitudes. This is a common mistake. Please remove all comparisons and rephrase all relevant discussions.

**Response**:

Thanks for your kind comment and reminder. We revised and rephrased our article accordingly.

3. In the Limitation section, please discuss briefly direction of bias and how they may affect your conclusion.

**Response**:

Thanks for your kind comment and reminder. We took your recommendation and revised our article accordingly.
